# Portal Fibroblasts: A Hidden Driver of Cholestasis-Associated Liver Cancer

**DOI:** 10.1016/j.jcmgh.2026.101798

**Published:** 2026-04-30

**Authors:** Sadam H. Bhat, Ekihiro Seki

**Affiliations:** Karsh Division of Gastroenterology and Hepatology, Department of Medicine, Cedars-Sinai Medical Center, Los Angeles, California; Karsh Division of Gastroenterology and Hepatology, Department of Medicine, Cedars-Sinai Medical Center, Los Angeles, California; Department of Biomedical Sciences, Cedars-Sinai Medical Center, Los Angeles, California

Chronic cholestatic liver diseases, including primary biliary cholangitis and primary sclerosing cholangitis create a continued fibroinflammatory environment that predisposes the liver to malignancies such as hepatocellular carcinoma (HCC) and cholangiocarcinoma.^1^ Although activated hepatic stellate cells (HSCs) have long been recognized as the primary drivers of liver fibrosis, the contribution of portal fibroblasts, a distinct stromal population located within the portal tract, remained understudied. Activated portal fibroblasts (aPFs) can be distinguished from HSCs by their expression of markers such as Thy1, mesothelin (Msln), and Mucin16 (Muc16), along with additional proteins including CD34, Gremlin, Fibulin2, and Col15a. Functionally, the Msln–Muc16–Thy1 signaling axis modulates transforming growth factor-β–mediated responses in aPFs, and disruption of this pathway limits Smad2/3-dependent activation, thereby reducing cholestatic fibrosis.^2^

In this issue of *Cellular and Molecular Gastroenterology and Hepatology*, Sakane and colleagues^3^ provide compelling evidence that this signaling axis in aPFs serves as a key regulator rather than a passive component of cholestatic fibrosis and HCC, particularly in the aged female liver. Using the Mdr2^−/−^ mouse model, the authors made several key observations. First, they confirm a striking sexual dimorphism, with aged female mice developing approximately 4-fold more tumors than males. Secondly, genetic deletion of Msln or Muc16, but notably not Thy1, significantly reduces fibrosis, inflammation, ductular reaction, and HCC burden, underscoring the crucial role of this pathway in disease advancement. Of note, these effects are mediated by stromal mechanisms, as tumor cells themselves do not express Msln or Muc16.

A key unique mechanistic development of this study is the discovery of paracrine communication between aPFs and hepatocytes. The authors found that matrix metalloproteinase-3 (MMP3) secreted by aPFs acts as an inhibitor of hepatocyte regeneration. Specifically, MMP3 promotes cleavage of the hepatocyte growth factor receptor c-Met on hepatocytes, thereby attenuating hepatocyte growth factor–dependent proliferative signaling. Conversely, aPFs lacking Msln or Muc16 exhibit reduced MMP3 expression, resulting in preserved c-Met signaling, improved hepatocyte proliferation, and decreased cellular senescence, a process closely associated with liver tumorigenesis through the senescence-associated secretory phenotype. This finding suggests that aPFs are not just passive matrix producers, but active players influencing hepatocyte health and cancer progression.

This study provides important conceptual insight, further supporting the emerging view that liver myofibroblasts are a heterogeneous population.^4^ The functional difference between activated HSCs and aPFs is particularly notable; although aPFs contribute nominally to cancer-associated fibroblasts within established tumors, they play a significant role in shaping the preneoplastic microenvironment. These observations suggest that targeting aPFs may repersent a potential strategy for tumorigenesis rather than as a treatment for advanced HCC, particularly in high-risk populations. This makes it a potential strategy for high-risk patients with primary sclerosing cholangitis/primary biliary cholangitis.

Furthermore, the increased susceptibility of aged female Mdr2^−/−^ mice supports previous reports of sexual dimorphism in this model,^5^ although the underlying mechanisms remain incompletely understood. This study establishes a foundation for future investigations into the interplay between hormonal factors and Msln–Muc16 signaling in aPFs.

Looking at the translational perspective, targeting Msln corresponds to a promising therapeutic opportunity. As a cell-surface protein, Msln has already been explored in clinical settings, including immunotoxin and chimeric antigen receptor T-cell–based approaches for mesothelioma and pancreatic cancer.^6^^,^^7^ The authors previously demonstrated that anti-Msln antibodies can attenuate cholestatic fibrosis, further supporting their therapeutic relevance.^2^ The present study extends this framework by implicating Msln inhibition as a potential strategy for preventing HCC. However, careful evaluation of safety will be essential, as Msln expression in nonhepatic mesothelial tissues raises the possibility of on-target, off-tumor toxicity.

Taken together, Sakane and colleagues successfully unmask the aPF as central regulators of both fibrosis and tumor development in cholestatic liver disease. By identifying the Msln–Muc16–MMP3–c-Met axis, they introduce a new conceptual framework understanding how periportal fibroblasts regulate distant hepatocyte function and the progression to HCC. This finding opens the door to a novel class of antifibrotic and cancer-preventive therapies targeting the portal fibroblast, moving beyond the traditional focus on HSCs and offering new hope for patients with age-related cholestatic liver disease and HCC.
